# Intervention effect of *Malus pumila* leaf flavonoids on senna‐induced acute diarrhea in BALB/c mice

**DOI:** 10.1002/fsn3.1549

**Published:** 2020-04-05

**Authors:** Ruokun Yi, Yu Tian, Fang Tan, Wenfeng Li, Jianfei Mu, Xingyao Long, Yanni Pan, Xin Zhao

**Affiliations:** ^1^ Chongqing Collaborative Innovation Center for Functional Food Chongqing University of Education Chongqing China; ^2^ Chongqing Engineering Research Center of Functional Food Chongqing University of Education Chongqing China; ^3^ Chongqing Engineering Laboratory for Research and Development of Functional Food Chongqing University of Education Chongqing China; ^4^ Department of Critical Care Medicine the First Affiliated Hospital of Chengdu Medical College Chengdu China; ^5^ Department of Public Health Our Lady of Fatima University Valenzuela Philippines; ^6^ School of Life Science and Biotechnology Yangtze Normal University Chongqing China; ^7^ Department of Food Science and Biotechnology Cha University Seongnam South Korea

**Keywords:** acute diarrhea, aquaporin, expression, flavonoid, *Malus pumila* leaf

## Abstract

The *Malus pumila* leaves are used as a kind of tea drink in China, and there are abundant flavonoids in the leaves of *Malus pumila*. In this study, BALB/c mice received senna extract solution via gavage, which induced acute diarrhea, and the interventive effect of *Malus pumila* leaf flavonoids (MPLF) was observed. The results showed that MPLF decreased the diarrhea index, and MPLF also decreased the capillary permeability in the abdominal cavity of mice. The result of serum cytokine detection indicated that MPLF decreased the levels of inflammatory cytokines, including interleukin 6 (IL‐6), interleukin 12 (IL‐12), and tumor necrosis factor alpha (TNF‐α). The results of quantitative polymerase chain reaction (qPCR) indicated that diarrhea decreased the mRNA and protein expression of aquaporin‐3 (AQP3) in the jejunum and aquaporin‐4 (AQP4) in the ileum, which were inhibited by MPLF. By high performance liquid chromatography (HPLC), MPLF mainly contains 10 flavonoids, which are rutin, hyperoside, isoquercitrin, taxifolin, quercitrin, hesperidin, myricetin, baicalin, neohesperidin dihydrochalcone and quercetin, the synergistic effect of these components plays an antidiarrhea role in MPLF. Therefore, MPLF achieved good antidiarrheal effect, which was better than that of the commonly used montmorillonite powder at the same concentration. MPLF is a natural raw material for functional food with an antidiarrheal effect.

## Graphical abstract

Malus pumila leaf flavonoids inhibited senna‐induced acute diarrhea in BALB/c mice through up‐regulation AOP3 mRNA and protein expression in jejunum tissue and AQP4 expression in ileum tissue.

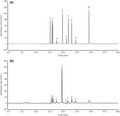

## 1 INTRODUCTION

*Malus pumila* is a kind of apple plant in *Rosaceae*, whose fruit is the apple that we often eat (Holloway, 1973). The leaves of the wild *Malus pumila* are also used in food processing. The leaves of wild *Malus pumila* are not only processed into drinks as tea like drinks, but also used as natural preservatives in other foods because of their antiseptic and bacteriostatic effects (Li et al., 2019). In addition, the leaves of *Malus pumila* are also used as folk medicine to prevent diarrhea (Zhu et al., 2019). There are rich vitamins, amino acids and other beneficial substances in the leaves of *Malus pumila*. After rough processing, the yield of total flavonoids in the dried leaves of *Malus pumila* can be more than 3%, the flavonoids are rich chemical substances in the leaves of *Malus pumila* (Lu, Yang, & Xia, 2014; Zhang & Sun, 2015).

Diarrheal disease is a common and important public health problem, defined epidemiologically across the world, which severely affects people's normal life activities. At present, antibiotics and other drugs are used for the treatment of diarrheal diseases. The use of antibiotics will eventually cause drug resistance in the body, thus diminishing their therapeutic effect (Hornick, 1967; Wilson, Buchanan, Allan, & Tikoo, 2012). Another class of alternative antibiotics, mostly antihistamines, has strong side effects. Improper use will cause severe physiological side effects; therefore, their use is limited by various restrictions (Marshall 2000). The use of foods for the prevention and treatment of diarrhea can avoid other side effects on the body and offer high safety. Therefore, using plant‐derived natural active substances to prevent and treat diarrhea (or related diseases) has become one of the current research hotspots.

This study established a mouse diarrhea model, and the effect of *Malus pumila* leaf flavonoids (MPLF) on diarrhea was studied for the first time. The mechanism underlying the effect of MPLF was verified preliminarily by a molecular biology experiment. The results can promote the application of MPLF as a natural functional food for diarrhea treatment, which is conducive to the development and utilization of its biological activity.

## 2 MATERIALS AND METHODS

### 2.1 Extraction of MDLF

The dried *Malus pumila* leaves (Yichang Fengxiang Biotechnology Co., Ltd) were crushed, and then, 200 g of the powder was precisely weighed and placed in the beaker, and 70% ethanol (*v*/*v*) was added according to the liquid to material ratio of 20:1. The mixture was stirred for 3 hr in the 60°C water bath (HH‐W420, Changzhou Yitong Analytical Instrument Manufacturing Co., Ltd), and then, the extraction liquid was extracted and filtered. The liquid was passed through FL‐3 macroporous resin (Shanghai Yiji Biology Co., Ltd), and the liquid passing through the resin was evaporated to dryness by rotary evaporator (R‐1001‐VN, Zhengzhou Greatwall Scientific Industrial and Trade Co., Ltd) to obtain the extracted MDLF.

### 2.2 Senna decoction (diarrhea inducer)

100 g of dried senna leaves (without veins, Bozhou Jingwan Herbal Pieces Factory) was decocted in 100 ml boiling distilled water for 30 min. The residue was filtered to obtain the senna decoction at a concentration of 0.3 g/ml, stored at 4°C, and heated in a water bath at 25°C when used.

### 2.3 Animal experiment

Fifty BALB/c mice (25 male and 25 female, 6 weeks old, 22 ± 3 g) were purchased from Chongqing Medical University (Chongqing, China). The mice were randomly divided into five groups: normal group (Normal), model group (Model), montmorillonite powder group (MP), low‐concentration MPLF group (LMPLF), and high‐concentration MPLF group (HMPLF) (*n* = 10 each). The mice were housed in separate cages at 25°C and 12‐hr/12‐hr day/light cycle. Before the experiment, all mice were fasted for 12 hr to clear the stomach and intestine from food and feces. After 12 hr, 0.3 ml senna decoction was given to each mouse by gavage, with the exception of the normal group who received 0.3 ml distilled water instead. After 1 hr, the mice in both the normal and the model groups were given 0.3 ml distilled water by gavage, and mice in the MP group received montmorillonite powder by gavage at a concentration of 50 mg/kg. The mice in the LMPLF and HMPLF groups received MPLF at concentrations of 25 mg/kg and 50 mg/kg by gavage, respectively. All administrations lasted for three days. Each mouse was placed in a cage with absorbent paper that was replaced hourly to record the defecations. Each index was observed for consecutive 5 hr. The diameter of the loose stool on the absorbent paper was recorded, and loose stool was distinguished from dry stool based on whether the stool was shaped or whether water‐circle pollution had formed on the absorbent paper. Normal dry stool had a granular shape and did not adhere to the absorbent paper; loose stool did not have a granular shape, adhered to the absorbent paper, and formed a circular stain. The data were observed and recorded daily for three consecutive days. The loose stool rate was calculated as the ratio of the times of defecations per mouse to the total number of defecations, where each granule or pile of defecations on the filter paper was counted as one (Zhang et al., 2010). The loose stool grade was calculated as the diameter (cm) of the area of the contamination on the filter paper: grade 1 (<1 cm), grade 2 (1–1.9 cm), grade 3 (2–3 cm), and grade 4 (>3 cm). When counting, the grade of each pile of loose stool was counted one by one, and then, all grades were summed and divided by the number of stool circles to calculate the average grade of stools. The diarrhea index was calculated as the product of the average grade of loose stools. The protocol for these experiments was approved by the Ethics Committee of Chongqing Collaborative Innovation Center for Functional Food (201904021B), Chongqing, China.

### 2.4 Detection of abdominal capillary permeability

Fifty mice (25 male and 25 female, 6 weeks old, Chongqing Medical University, Chongqing, China) were grouped and treated before the experiment as described above. On the third day, 1 hr after gavage, the mice in each group were injected with Evans Lan saline (Beijing Leagene Biotechnology Co., Ltd) solution at a concentration of 2 g/100 ml via the tail vein at a dose of 10 ml/kg. The mice with successful injection were immediately intraperitoneally injected with 0.2 ml of 0.6% acetic acid. After 20 min, blood was collected from the eyeballs and centrifuged at 2,504 *g* for 10 min at 4°C (iCEN‐24R high speed freezing centrifuge, Hangzhou Allsheng Instruments Co., Ltd, Hangzhou, Zhejiang, China) to obtain serum, which was stored at −80°C after subpackaging. The abdominal cavity was opened and was rinsed several times with 5 ml normal saline. The cleaning solution was collected and centrifugated at 157 *g* for 5 min (iCEN‐24R high speed freezing centrifuge, Hangzhou Allsheng Instruments Co., Ltd). Its absorbance was measured at 590 nm (Evolution™ 350 ultraviolet visible spectrophotometer, Thermo Fisher Scientific, Inc.).

### 2.5 Measurement of serum cytokine levels

Mouse housing and feeding were identical as above. Before blood collection, mice were fasted for 12 hr. Whole blood was obtained from eyeballs and centrifugated at 22,539 *g* for 10 min under 4°C to obtain serum. Serum cytokine levels were measured by kits (Nanjing Jiancheng Bioengineering Institute).

### 2.6 mRNA‐related gene expression measured by qPCR

The dissected jejunum and ileum (about 100 mg) were placed in a centrifuge tube and homogenized with 1 ml TRIzol (Thermo Fisher Scientific, Inc.). After centrifugation with 400 ml isopropanol at 33,678 *g* for 20 min, the supernatant was discarded and the extracted RNA was obtained. 500 μL of DEPC water was added to the extracted RNA, then centrifuged (33,678 *g*, 20 min), the supernatant discarded, and 20 μl DEPC water was added to the mix. 1 μl RNA, 1 μl Oligo Primer (Thermo Fisher Scientific, Inc.), and 1 μl water were added to the centrifuge tube, then centrifuged, placed in a PCR instrument, and mixed, at 65°C for 5 min. Then, 4 μl 5 × Reaction Buffer, 1 μl Ribdock RNase Inhibitor (20 μ/μl) and 2 μl 100 mmol/L dNTP mix (Thermo Fisher Scientific, Inc.) were mixed and added to the centrifuge tube. 1 μl Revert Aid Mrmvdv RT (Thermo Fisher Scientific, Inc.) was added to the mix, centrifuged, and mixed with the PCR reagent (Stepone Plus, Thermo Fisher Scientific, Inc.). The parameters were set to 42°C for 60 min and 70°C for 5 min to obtain the sample cDNA solution. 10 μl cDNA solution, 10 μl master, 1 μl forward primer, 1 μl reverse primer (Table 1), 7 μl ddH_2_O, and 1 μl cDNA solution were mixed and then tested. Amplification conditions were as follows: denaturation at 95°C for 15 min, annealing at 60°C for 1 hr, extension at 95°C for 15 min, and 40 cycles. The relative expression of each gene was calculated by the 2^‐ΔΔCT^ method, using GAPDH as internal reference gene (Long, Pan, & Zhao, 2018).

**Table 1:** Sequences of primers used in the qPCR assay

Gene Name	Sequence
*AQP3*	Forward: 5′‐GCCTCCCTTATCGTGTGTGTGC‐3′
	Reverse: 5′‐AGGTGGCAGCCGATCAGC‐3′
*AQP4*	Forward: 5′‐GCTGTGATTCCAAACGGACTGATG‐3′
	Reverse: 5′‐CTGACTCCTGTTGTCCTCCACCTC‐3′
*GAPDH*	Forward: 5'‐AGGTCGGTGTGAACGGATTTG‐3'
	Reverse: 5'‐GGGGTCGTTGATGGCAACA‐3'

### 2.7 Protein‐Related Expression Measured by Western Blot

A portion of the jejunum and ileum tissue (100 mg) was homogenized with 1 ml of RIPA and 10 μL of PMSF (Easy Bio, Beijing, China) at 22,539 *g*, then lysed at 4°C for 5 min, and centrifuged at 22,539 *g* and 4°C for 15 min. The protein in the middle layer was quantified using a BCA kit (Bio‐Rad Laboratories). Each sample was diluted to 50 µg/ml, then heated at 100°C for 5 min with a sample buffer at a 4:1 ratio, and cooled on ice for 5 min. Mixing acrylamide, resolving buffer, stacking buffer, distilled water, 10% of APS, and TEMED were added at a given ratio to prepare SDS‐PAGE running gel (Thermo Fisher Scientific, Inc.) and stacking gel, and loaded for run. Prestained protein ladder and samples were injected into the well of the plate, followed by SDS‐PAGE vertical gel electrophoresis for 50 min. The PVDF membrane was activated by methanol for 1 min and then sealed with 1 × TBST containing 5% skimmed milk for 1 hr. After sealing, the PVDF membrane was cleaned with 1 × TBST (Solarbio Life Sciences) and incubated with primary antibody (Thermo Fisher Scientific, Inc.) at 25°C for 2 hr. The membrane was further incubated with the secondary antibody (Thermo Fisher Scientific, Inc.) at 25°C for 1 hr after being cleaned with 1 × TBST for five times. Finally, the PVDF membrane was sprayed with Supersignal West Pico PLUS and then placed in an imaging system (Tanon 5200, Tanon Science and Technology Co., Ltd) for observation (Liu, Tan, Liu, Yi, & Zhao, 2019).

### 2.8 High performance liquid chromatography (HPLC)

*Malus pumila* leaf flavonoids was dissolved in DMSO to prepare a sample solution with a concentration of 10 mg/ml, then diluted with 50% methanol to prepare a solution to be tested with a concentration of 2 mg/ml, then the solution to be tested is filtered by 0.22 μm organic filter membrane and tested on the machine. The detection conditions were chromatographic column: Thermo Scientific accucore C18 (4.6 mm × 150 mm, 2.6 μm), gradient elution mobile phase B was acetonitrile, mobile phase A was 0.5% glacial acetic acid aqueous solution, flow rate was 0.5 ml/min, column temperature was 35°C, detection wavelength was 360 nm, and injection volume was 5 μL (UltiMate3000 HPLC System, Thermo Fisher Scientific, Inc.). The gradient elution conditions were shown in Table 2.

**Table 2:** Gradient elution conditions of mobile phase

Time (min)	Mobile phase A (%)	Mobile phase B (%)
0	88	12
30	60	40
35	0	100
40	0	100

### 2.9 Data analysis

Experimental data were analyzed by GraphPad prism 7 (GraphPad Software) ANOVA was used, and Duncan's multiple range test was used to assess the significance level. The data of three experiments from each group were averaged.

## 3 RESULTS AND DISCUSSION

### 3.1 Effect of MPLF on acute diarrhea

As shown in Table 3, the diarrhea index of the normal group was 0 within three days, indicating that the housing environment and the feed was good and did not cause diarrhea. The difference in diarrhea index between model group and normal group was statistically significant (*p* < .05), indicating successful experimentation. Compared with the model group, the diarrhea indexes of the MP, LMPLF, and HMPLF groups were statistically significant (*p* < .05). The index of the HMPLF group was lower and closer to that of the normal group, which significantly differed from the other two groups (*p* < .05). This indicated that HMPLF exerted an intervention effect on diarrheal disease caused by senna, which was better than that of MP.

**Table 3:** Effect of *Malus pumila* leaf flavonoids on diarrhea index in mice caused by senna leaf decoction (*N* = 10)

Group	Diarrhea index		
	1st day	2nd day	3rd day
Normal	0.00 ± 0.00^e^	0.00 ± 0.00^e^	0.00 ± 0.00^e^
Model	1.76 ± 0.22^a^	1.33 ± 0.19^a^	1.12 ± 0.12^a^
MP	0.69 ± 0.07^c^	0.57 ± 0.06^c^	0.41 ± 0.05^c^
LMPLF	1.21 ± 0.12^b^	0.92 ± 0.06^b^	0.73 ± 0.05^b^
HMPLF	0.42 ± 0.06^d^	0.33 ± 0.05^d^	0.20 ± 0.04^d^

Note

Values presented are the mean ± standard deviation.

Mean values with different letters (a‐d) in the same column are significantly different (*p* < .05) according to Duncan's new MRT. Normal: untreated mice, Model: mice treated with 3 ml senna leaf decoction, MP: mice treated with 50 mg/kg montmorillonite powder, LMPLF: mice treated with 25 mg/kg *Malus pumila* leaf flavonoids, HMPLF: mice treated with 50 mg/kg *Malus pumila* leaf flavonoids.

Abbreviations: HMPLF, high‐concentration MPLF group; LMPLF, low‐concentration MPLF group; MP, montmorillonite powder group.

Diarrhea is one of the common diseases in the digestive system and can be divided into acute and chronic diarrhea according to the duration. Acute diarrhea is most often caused by bacterial infections of the intestinal tract. The pathogenic factors of chronic diarrhea are diverse, and it can be caused by infection or intestinal inflammation. The occurrence of severe and long‐term diarrhea symptoms can lead to dehydration, thus resulting in electrolyte disorders, which severely affecting health. The main symptoms of diarrhea include increases in the times of bowel movements per day and feces with paste‐like or even water‐like consistency. Senna is a stimulant laxative, stimulates intestinal peristalsis through the intestinal mucosa and nerves, and is a commonly used laxative in the clinic context (Lu & Cui, 2007). Therefore, this study used senna as diarrhea inducer for the mouse model.

It has been reported that the use of the diarrhea index to reflect the degree of loose stool in diarrhea models not only considers the change of dilution volume, but also the quality factor. It is more comprehensive and objective than both indexes of dilution rate and dilution grade alone and enables their comparison. Statistical analysis has also confirmed that the diarrhea index of mice is normally distributed and can be statistically processed according to relevant parameters. Previous experiments also showed no difference in diarrhea indexes between groups and good reproducibility in mice (Gu et al., 2019). The present study proved that the diarrhea index in the MPLF‐treated group was significantly lower than in the model group (*p* < .05).

### 3.2 Effect of MPLF on capillary permeability in the abdominal cavity of sense‐induced mice

As shown in Table 4, the absorbance of peritoneal lavage fluid of normal mice was lowest in the standard range, indicating good initial condition of the mice. Compared with the normal group, the model group had the highest absorbance of the abdominal capillary permeability index and showed a significant difference (*p* < .05), indicating successful diarrhea modeling in these mice. The absorbance values in the HMPLF and LMPLF groups were significantly lower compared with the model group. This indicated that MPLF exerted a significant intervention effect on senna‐induced diarrhea, and the effect in the HMPLF group was also higher than that of the MP group.

**Table 4:** Effect of *Malus pumila* leaf flavonoids on mouse peritoneal capillary permeability induced by senna leaf decoction (*N* = 10)

Group	Absorbance value (OD_590_)
Normal	0.662 ± 0.006^e^
Model	1.041 ± 0.010^a^
MP	0.792 ± 0.007^c^
LMPLF	0.896 ± 0.005^b^
HMPLF	0.705 ± 0.005^d^

Note

Values presented are the mean ± standard deviation.

Mean values with different letters (a‐d) in the same column are significantly different (*p* < .05) according to Duncan's new MRT. Normal: untreated mice, Model: mice treated with 3 ml senna leaf decoction, MP: mice treated with 50 mg/kg montmorillonite powder, LMPLF: mice treated with 25 mg/kg *Malus pumila* leaf flavonoids, HMPLF: mice treated with 50 mg/kg *Malus pumila* leaf flavonoids.

Abbreviations: HMPLF, high‐concentration MPLF group; LMPLF, low‐concentration MPLF group; MP, montmorillonite powder group.

Inflammation of the body causes increased vascular permeability. After diarrhea in mice had been induced by senna, inflammation would occur in the abdominal cavity and intestinal tract, correspondingly enhancing the capillary permeability in the abdominal cavity (Zhang, Chen, Wang, Zhu, & Mo, 2003). The results showed that MPLF could preintervene with this abnormal increase in peritoneal capillary permeability in mice. Thus, MPLF is suggested to achieve antidiarrheal effect via anti‐inflammation.

### 3.3 Effect of MPLF on serum IL‐6, IL‐12, and TNF‐α contents in mice

As shown in Table 5, compared with the normal group, the serum levels of IL‐6, IL‐12, TNF‐α in the model group were up‐regulated and showed a significant difference compared with the normal group (*p* < .05). Compared with the model group, the expression levels of IL‐6, IL‐12, and TNF‐α were significantly down‐regulated in the HMPLF, LMPLF, and MP groups (*p* < .05). The HMPLF group had significant down‐regulation of various indexes, which differed significantly from the expression levels of the model group. This indicated that MPLF had the ability to prevent senna‐induced diarrhea. The higher the concentration, the more obvious the effect.

**Table 5:** Effect of *Malus pumila* leaf flavonoids on serum cytokine levels of mice induced by senna leaf decoction (*N* = 10, μg/L)

Group	IL‐6	IL‐12	TNF‐α
Normal	45.33 ± 1.62^e^	142.97 ± 6.23^e^	30.63 ± 2.52^e^
Model	171.06 ± 5.82^a^	782.19 ± 12.56^a^	139.55 ± 7.80^a^
MP	88.36 ± 6.11^c^	412.01 ± 14.32^c^	74.86 ± 5.29^c^
LMPLF	120.59 ± 7.08^b^	556.47 ± 10.86^b^	98.71 ± 6.22^b^
HMPLF	63.07 ± 5.09^d^	279.26 ± 11.57^d^	51.52 ± 4.97^d^

Note

Values presented are the mean ± standard deviation.

Mean values with different letters (a‐d) in the same column are significantly different (*p* < .05) according to Duncan's new MRT. Normal: untreated mice, Model: mice treated with 3 ml senna leaf decoction, MP: mice treated with 50 mg/kg montmorillonite powder, LMPLF: mice treated with 25 mg/kg *Malus pumila* leaf flavonoids, HMPLF: mice treated with 50 mg/kg *Malus pumila* leaf flavonoids.

Abbreviations: HMPLF, high‐concentration MPLF group; LMPLF, low‐concentration MPLF group; MP, montmorillonite powder group.

Inflammatory cytokines are various cytokines involved in the inflammatory response. Cytokines, such as IL‐6, IL‐12, and TNF‐α, play a major role. TNF‐α is the earliest and most important inflammatory mediator during the inflammatory response, which activates neutrophils and lymphocytes, increases the permeability of vascular endothelial cells, regulates the metabolic activity of other tissues, and promotes both synthesis and release of other cytokines (Teng et al., 2015). IL‐6 can induce B cells to differentiate, produce antibodies, and induce T cells to activate proliferation and differentiation. Consequently, IL‐6 participates in the immune response of the body and acts as a promoter of the inflammatory response (Gabr et al., 2011). IL‐12 can stimulate the chemotaxis of neutrophils, T lymphocytes, and eosinophils, promote neutrophil degranulation, release elastase, damage endothelial cells, cause microcirculation blood flow stasis and tissue necrosis, and damage organ function (Ishihara & Hirano 2002). All of these are expression factors indicating inflammation of the body. The results of the present study showed that the expressions of IL‐6, IL‐12, and TNF‐α in the MPLF group were significantly lower than in the model group (*p* < .05), indicating that MPLF could prevent peritoneal inflammation caused by senna. This verifies that MPLF can achieve an antidiarrheal effect by anti‐inflammation.

### 3.4 mRNA and protein expression of *AQP3* and *AQP4* in the jejunum and ileum tissue

**Figure 1:**
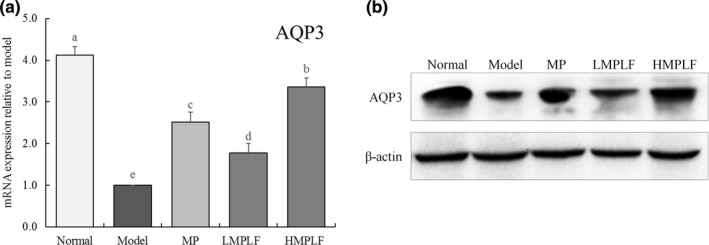
mRNA and protein expression of AQP3 in jejunal tissue of mice. Values presented are the mean ± standard deviation. ^a‐e^ Mean values with different letters in the bars are significantly different (*p* < .05) according to Duncan's new MRT. Normal: untreated mice, Model: mice treated with 3 ml senna leaf decoction, MP: mice treated with 50 mg/kg montmorillonite powder, LMPLF: mice treated with 25 mg/kg *Malus pumila* leaf flavonoids, HMPLF: mice treated with 50 mg/kg *Malus pumila* leaf flavonoids

**Figure 2:**
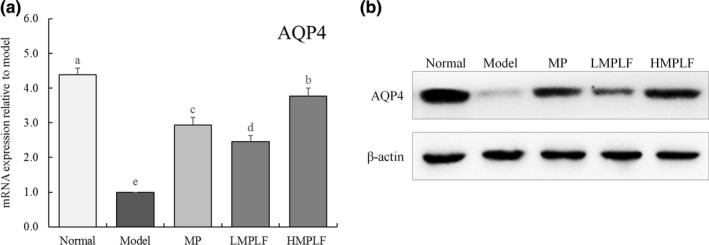
mRNA and protein expression of AQP4 in ileal tissue of mice. Values presented are the mean ± standard deviation. ^a‐e^ Mean values with different letters in the bars are significantly different (*p* < .05) according to Duncan's new MRT. Normal: untreated mice, Model: mice treated with 3 ml senna leaf decoction, MP: mice treated with 50 mg/kg montmorillonite powder, LMPLF: mice treated with 25 mg/kg *Malus pumila* leaf flavonoids, HMPLF: mice treated with 50 mg/kg *Malus pumila* leaf flavonoids

As shown in Figures 1 and 2, compared with the normal group, the expression of *AQP3* in the jejunum and *AQP4* in the ileum decreased significantly in the model group (*p* < .05). This showed that the expression of *AQP3* and *AQP4* decreased, the water absorption capacity of cells decreased, and cells severely lost water in the diarrhea state. In the HMPLF, LMPLF, and MP groups, compared with the model group, the expression levels of *AQP3* and *AQP4* in the treatment group were up‐regulated to different degrees, and each group showed significant differences (*p* < .05). The indexes of the HMPLF group were significantly up‐regulated (*p* < .05), indicating that MPLF could maintain the down‐regulation of AQP3 and AQP4 proteins, and intervene with senna‐induced diarrhea in mice. Moreover, the effect of MPLF was better than that of MP at the same concentration.

The main component of an organism is aqueous solution composed of water and various ions that enter and exit channels in the cell membrane to realize cellular functions. Aquaporin, a protein located on the cell membrane, constitutes a "pore" and controls the entry and exit of water in the cell, similar to a "water pump.” It is an important channel for the material exchange inside and outside of cells, and its family is composed of AQP0, AQP1, AQP2, AQP3, and AQP4 proteins (Zhang et al., 2017). AQP3 and AQP4 are located in the kidney and are related to water reabsorption. When cells are dysfunctional, senescent, or water‐deficient, the expressions of the AQP3 and AQP4 genes are down‐regulated, which leads to decreased water absorption capacity of cells, and a state of water shortage (Laforenza, 2012). This study also indicated that in the model group, compared with the normal group, the mRNA expression of the AQP3 and AQP4 genes was significantly decreased, and the expressions of the AQP3 and AQP4 genes in the MPLF group were up‐regulated compared with the model group. This indicated that MPLF could inhibit the disorder of cellular regulation caused by diarrhea, and the effect was better than that of MP.

### 3.5 Composition of MPLF

**Figure 3:**
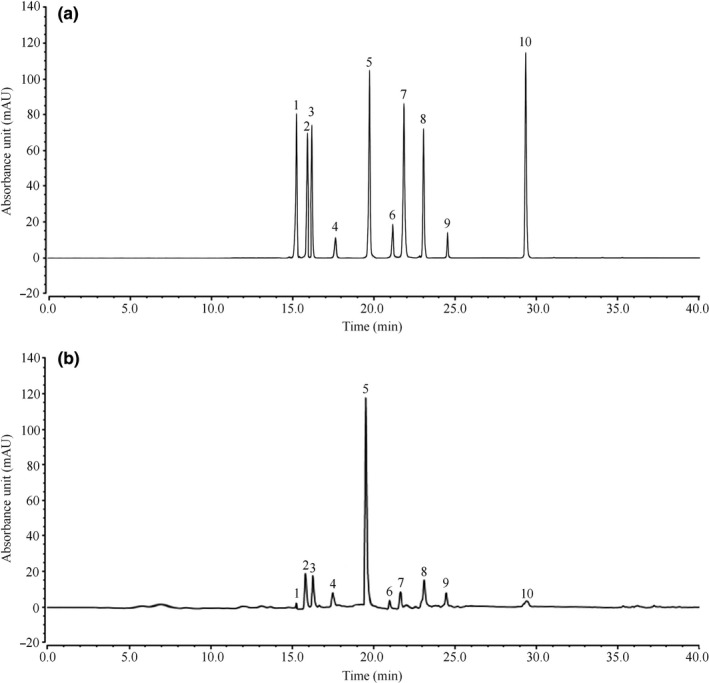
High performance liquid chromatography chromatograms of (a) standard products and (b) TRE. 1: rutin, 2: hyperoside, 3: isoquercitrin, 4: taxifolin, 5: quercitrin, 6: hesperidin, 7: myricetin, 8: baicalin, 9: neohesperidin dihydrochalcone, 10: quercetin

The results of component analysis of MPLF showed that it mainly contained 10 compounds, namely rutin, hyperoside, isoquercitrin, taxifolin, quercitrin, hesperidin, myricetin, baicalin, neohesperidin dihydrochalcone, and quercetin (Figure 3). Through quantitative analysis, it could be seen that the compounds with high contents in MPLF included hyperoside, isoquercitrin, and quercitrin (Table 6).

**Table 6:** Linear relationship, regression equation and content of chemical components in TRE

Chemical composition	Linear equation	R^2^	Content (mg/g)
Rutin	*y* = 5.6487*x*−3.9033	0.9993	1.45 ± 0.02
Hyperoside	*y* = 5.2655*x*−2.7604	0.9991	84.77 ± 0.04
Isoquercitrin	*y* = 5.3088*x*−3.7092	0.9990	56.04 ± 0.15
Taxifolin	*y* = 1.0262*x*−0.521	0.9992	0.89 ± 0.03
Quercitrin	*y* = 7.1445*x*−4.7704	0.9991	226.25 ± 1.22
Hesperidin	*y* = 1.1892*x*−0.6226	0.9990	9.35 ± 0.05
Myricetin	*y* = 7.5639*x*−5.4068	0.9992	1.79 ± 0.04
Baicalin	*y* = 7.3687*x*−5.1281	0.9990	14.09 ± 0.06
Neohesperidin dihydrochalcone	*y* = 0.8671*x*−0.3938	0.9990	1.29 ± 0.03
Quercetin	*y* = 8.3117*x*−5.2751	0.9990	12.66 ± 0.11

Rutin, hyperoside, isoquercitrin, taxifolin, quercitrin, hesperidin, myricetin, baicalin, neohesperidin dihydrochalcone, and quercetin are common flavonoids in plants, all of which have good biological activities and have positive effects on many diseases (Selloum et al., 2003; Ku et al., 2015; Li, Zhang, Liu, Liu, & Dong, 2016; Matsuda et al., 2007; Ma, Luo, Jiang, & Liu, 2016; Yan & Hu, 2004; Tong, Zhou, Wang, Yang, & Cao, 2009; Yan et al., 2017; Bar, Borrego, Benavente, Castillo, & Rio, 1990; Rivera, Morón, Sánchez, Zarzuelo, & Galisteo, 2008). All of these 10 compounds have good anti‐inflammatory effects (Bar et al., 1990; Ku et al., 2015; Li et al., 2016; Ma et al., 2016; Matsuda et al., 2007; Rivera et al., 2008; Tong et al., 2009; Yan et al., 2017; Yan & Hu, 2004). Hyperoside and neohesperidin dihydrochalcone have protective effects on gastrointestinal mucosa and can regulate gastrointestinal function (Bar et al., 1990; Ku et al., 2015). Taxifolin and hesperidin have bacteriostatic effect, which can kill harmful bacteria in the intestine and keep the intestine healthy (Matsuda et al., 2007; Yan et al., 2017). Baicalin not only has bacteriostatic effect, but also has the effect of stopping diarrhea and vomiting (Yan et al., 2017). MPLF contained these 10 compounds, which directly or cooperatively form the biological activities of MPLF, and made it produce the activity of antidiarrhea.

## 4 CONCLUSIONS

The intervention effect of MPLF on diarrhea was tested in senna‐induced mice with acute diarrhea. The results showed that MPLF effectively controlled the abnormal increase in vascular permeability by alleviating the inflammatory response. It could also regulate aquaporins to prevent cell dysfunction and protect intestines, thus acting as an antidiarrheal agent. Therefore, MPLF was identified as a natural substance that can be utilized and marketed for its antidiarrheal effect. As a basic preclinical study, the antidiarrheal effect of MPLF in animals was investigated for the first time. Further in‐depth studies investigating the in vivo mechanism of MPLF are needed to achieve practical application of MPLF through human clinical trials.

## CONFLICT OF INTEREST

The authors of this manuscript state that they do not have conflict of interest to declare.

## ETHICAL APPROVAL

This study was approved by the Ethics Committee of Chongqing Collaborative Innovation Center for Functional Food (201906009B), Chongqing, China.
